# Negative and Reversible Magnetorheological Response for Magnetic Rubbers

**DOI:** 10.3390/gels11120969

**Published:** 2025-12-02

**Authors:** Rentaro Kanamori, Tomoya Sako, Hiroaki Okazaki, Mika Kawai, Tetsu Mitsumata

**Affiliations:** 1 Graduate School of Science and Technology, Niigata University, Niigata 950-2181, Japan; 2 Molten Corporation, Hiroshima 733-0036, Japan

**Keywords:** stimuli-responsive gel, magnetic responsive gel, rubber, soft material

## Abstract

A material exhibiting a reversible decrease in elastic modulus upon application of a magnetic field has been successfully developed for the first time. The material is a composite of natural rubber and carbonyl iron with a particle diameter of 8.3 μm. The storage modulus in the absence of magnetic field is 155 kPa and it decreases to 89.5 kPa by applying a magnetic field of 500 mT. The rubber composite underwent reversible changes in the dynamic modulus even after 30 cycles of on-off switching of the magnetic field.

## 1. Introduction

Stimuli-responsive soft materials that change their physical properties in response to stimuli such as light, temperature, and pH [1,2,3,4,5,6,7,8,9,10] have been widely investigated since the past decade. Magnetic soft materials such as magnetic gel, magnetic elastomer, and magnetic rubber, are composite materials consisting of polymer matrix and magnetic substance such as magnetic particles or magnetic fluids. The physical properties of magnetic soft materials alter in response to magnetic fields [11,12,13,14,15,16,17,18,19,20]. Magnetic soft materials are attracting significant attention as next-generation actuators since they instantly and dramatically change not only their mechanical properties but also other physical properties when a magnetic field is applied [21,22,23,24,25,26,27,28,29,30]. Physical property in response to magnetic fields presented here is mechanical property. The elastic modulus becomes high compared to the original modulus due to magnetic fields. This is called the magnetorheological (MR) effect. The main cause is that magnetic particles make contact points with by rearrangement and form a chain-like structure in the polymer matrix [31]. For details on the mechanism of the origin of the magnetorheological effect, it has been summarized in some recent reviews [32,33,34].

Most of the MR materials demonstrate positive changes in the elastic modulus by applying magnetic fields. However, there are some MR materials showing negative changes in the elastic modulus. Carrageenan magnetic gels demonstrate negative MR effect [35,36,37]. These negative MR effects are seen in soft materials in which magnetic particles are packed in high density and are caused by the destruction of the particle network of magnetic particles. However, the negative MR effect seen in these materials is irreversible, that is, the elastic modulus does not recover to the original modulus after removing the magnetic field. This is because once the contact points between particles collapse due to a magnetic field, the material itself cannot recover even after the magnetic field is removed. This means that the carrageenan gel lacks the restoring force that enables magnetic particles to pull-back to their original position. In the present study, reversible changes in the negative MR effect were succeeded to be realized. Probably, a restoring force is acting at the interface between the natural rubber and the magnetic particles, returning the magnetic particles to their original position. Industrially, there is strong demand for materials that change to a soft state only when a magnetic field is applied. However, materials that soften reversibly upon the application of a magnetic field have not yet been developed.

We introduce here a magnetic rubber recently developed that exhibits negative and reversible changes in the elastic modulus. The storage modulus for the magnetic rubber showing maximum changes was 155 kPa in the absence of magnetic field and it decreased to 89.5 kPa by applying a magnetic field of 500 mT. In the present system, even with only three samples, a negative change in elastic modulus can be clearly observed. The average value of the decrease in storage modulus due to the magnetic field for three samples was −6.52 ± 0.59 × 10^4^ Pa. Although the detailed mechanism of this very rare phenomenon is unclear in the present, the basic properties of the magnetic-field response of the dynamic modulus are presented in this paper and a possible mechanism is discussed.

## 2. Results and Discussion

**Figure 1:**
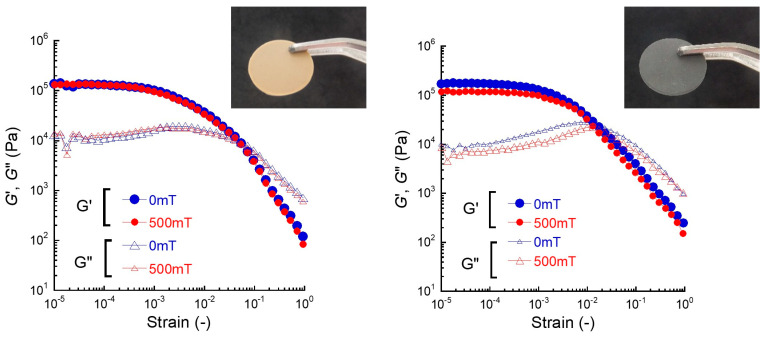
Impact of the magnetic field (500 mT) of the strain dependence of storage and loss moduli for natural rubber (**left**) and magnetic rubber (**right**) with a volume fraction of 0.11. Inset: Photographs of natural rubber and magnetic rubber.

Figure 1 shows the impact of the magnetic field on the strain dependence of storage for natural rubber and magnetic rubber with a volume fraction of 0.11. For the natural rubber, the storage modulus at 0 mT showed a constant value of 1.30 × 10^5^ Pa at strains below 3 × 10^−4^, i.e., linear viscoelastic region, and it significantly decreased with the strain. The loss modulus exhibited a broad peak at a strain of ~0.01 due to the nonlinear viscoelasticity. The strain at the point where *G*’ intersects *G*” was 0.04, suggesting that the viscosity is dominant at strains above this value. At 500 mT, the storage modulus nearly traced the storage modulus at 0 mT, suggesting that there was no magnetic field effect on the viscoelastic properties. For the magnetic rubber, the storage modulus at 0 mT showed a constant value of 1.71 × 10^5^ Pa at strains below 2 × 10^−4^, which was higher than that for the natural rubber due to the filling effect of magnetic particles. The mean values of storage modulus at 0 and 500 mT were 1.55 ± 0.10 × 10^5^ Pa and 8.95 ± 1.48 × 10^4^ Pa, respectively. The storage modulus decreases significantly with the strain, exhibiting behavior similar to natural rubber; however, it should be noted that its behavior differs somewhat from that of natural rubber. The strain at the point where *G*’ intersects *G*” was 0.02, which was slightly lower than that for the natural rubber. At 500 mT, the storage modulus at *γ* < 0.01 was 1.18 × 10^5^ Pa, which was clearly lower than that at 0 mT. However, these values were almost the same at *γ*~0.01 and they showed different values again at higher strains. This behavior is unique for magnetic rubbers, being different from the behavior of polysaccharide-based magnetic hydrogels [38] or polyurethane-based magnetic elastomers [39,40]. This decrease in the storage modulus by the strain is considered to be caused by the overlapping between the collapse of aggregations of magnetic particles and the strong nonlinearity of natural rubber, which will be discussed later. Although the magnetic response of mechanical properties is irreversible, there is a paper describing the negative magnetorheological response for magnetic soft materials by Sharma et al. [41], who pointed out the decrease in the elasticity is attributed to the destruction of local particle networks which are fragile to be broken by the application of small strain.

**Figure 2:**
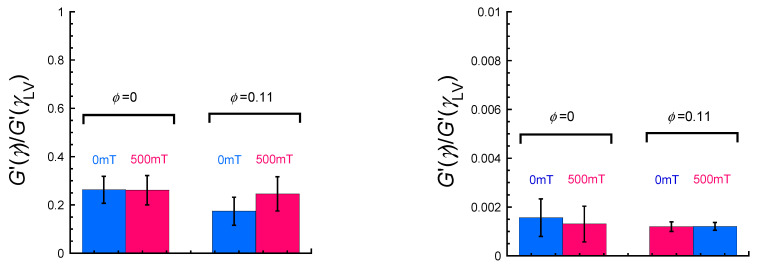
Effect of the magnetic field (500 mT) on the amplitude of the Payne effect *G*’(*γ*)/*G*’(*γ*_LV_) showing nonlinear viscoelasticity for natural rubber and magnetic rubber with a volume fraction of 0.11 at strain regions of 10^−5^ < *γ* < 10^−2^ (**left**) and 10^−5^ < *γ* < 1 (**right**).

Figure 2 exhibits the effect of the magnetic field on the amplitude of the Payne effect *G*’(*γ*)/*G*’(*γ*_LV_) [42] showing nonlinear viscoelasticity for natural rubber and magnetic rubber with a volume fraction of 0.11 at different regions of strain. The *G*’(*γ*) and *G*’(*γ*_LV_) represent the storage modulus at a certain strain and at strains of linear viscoelasticity, respectively. The amplitude of the Payne effect was defined as the storage modulus at certain strains, *G*’(*γ* = 10^−2^) or *G*’(*γ* = 1), relative to the storage modulus in the linear viscoelastic regime, *G*’(*γ* = 10^−5^). The value of *G*’(*γ*)/*G*’(*γ*_LV_) for the natural rubber was 0.26 ± 0.06 at 0 mT, which was same as that at 500 mT (0.26 ± 0.06). The value of *G*’(*γ*)/*G*’(*γ*_LV_) for the magnetic rubber was 0.17 ± 0.06 at 0 mT, and it increased to 0.25 ± 0.07 at 500 mT. These results strongly indicate that there is no structure in the natural rubber that is collapsed by the magnetic field, whereas there is a structure in the magnetic rubber that is collapsed by the magnetic field. In addition, the results also indicate that the structure for the magnetic rubber after collapsing by the magnetic field is the same as that of the natural rubber. At a strain of *γ* = 1, the value of *G*’(*γ*)/*G*’(*γ*_LV_) for the natural rubber was 0.0016 ± 0.001 at 0 mT, which was almost the same as that at 500 mT (0.0013 ± 0.001). It was also observed that the value of *G*’(*γ*)/*G*’(*γ*_LV_) for the magnetic rubber was 0.0012 ± 0.002 at 0 mT, which was almost the same as that at 500 mT (0.0012 ± 0.000). Thus, the *G*’(*γ*)/*G*’(*γ*_LV_) underwent similar values for all samples independently of magnetic particles. Therefore, this suggests that the structure of magnetic rubbers at a strain of 1 is close to a random structure without particle contact. According to our previous research [40], the value of *G*’(*γ*)/*G*’(*γ*_LV_) for polyurethane-based magnetic elastomers with same volume fraction of barium ferrite particles (*ϕ* = 0.12) at 0 mT was 0.60 ± 0.01 at the same strain (*γ*~0.01). Even barium ferrite, which exhibits significant aggregation within the polyurethane matrix, has values far exceeding that of the magnetic rubber studied here. As mentioned above, even natural rubber without magnetic particles exhibits a very low value at *γ* = 0.01 (=0.26). Therefore, it is evident that the strong nonlinearity of magnetic rubber is caused not only by the collapse of contact between magnetic particles but also by the strong nonlinearity of natural rubber itself. As a reason for the pronounced nonlinearity of rubber system without particle reinforcement, the essential mechanism for the Payne effect lies in the nature of entanglement network in the rubbery matrix, and the disentanglement of rubber molecular chains has been proposed in the literature [43,44,45,46].

**Figure 3:**
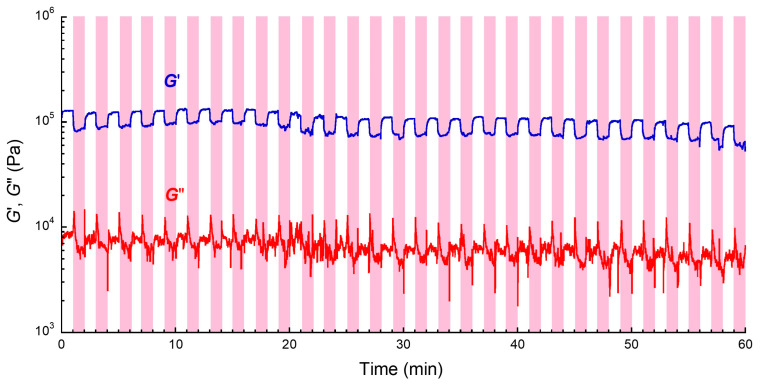
Magnetic-field response of storage and loss moduli at a strain of 10^−4^ for magnetic rubber with a volume fraction of 0.11. A magnetic field of 500 mT was applied in the colored area.

Figure 3 exhibits the magnetic-field response of storage modulus at a strain of 10^−4^ for magnetic rubber with a volume fraction of 0.11, obtained by on–off switching a magnetic field of 500 mT with 30 cycles, which is important for applications. If the polymer or particle network is broken, the waveform changes certainly from the second switching. In our experience, if there is no significant drop within 20 cycles, the MR effect is not degraded by switching the magnetic field for a larger number of cycles. The storage modulus for the magnetic rubber changed perfectly in response to the magnetic field even after the on–off switching of the magnetic field with 30 cycles. This strongly suggests that the structure of magnetic particles under the magnetic field is a quasi-stable structure even though the arrangement of magnetic particles is agitated by the switching of magnetic field. It is also found that no large change in the off-field modulus was seen even after the on–off switching. General in cross-linked materials exhibiting a positive MR effect, the off-field modulus gradually increases with the number of switching cycles [47]. This is due to the structural changes caused by the agglomeration of magnetic particles [38]. Surprisingly, the result of the magnetic rubber indicates that the arrangement of magnetic particles almost recovers to its original state after the magnetic field is switched off, which may be attributed to the adhesion between the magnetic particles and the matrix.

In order to evaluate the adhesion property between magnetic particles and the natural rubber, the degree of swelling was determined. The degree of swelling for natural rubber was 1.15 ± 0.02 and it for the magnetic rubber was 1.07 ± 0.01. Since the cross-linking density is same as these materials, this suggests that the magnetic particles make the rubber matrix restrict from swelling. This also indicates that strong interactions exist between magnetic particles and the matrix of natural rubber. Strong interactions between particles and the matrix increase the restoring force due to particle movement. This may be the origin of the reversibility of variable elasticity for magnetic soft materials with aggregative particles.

**Figure 4:**
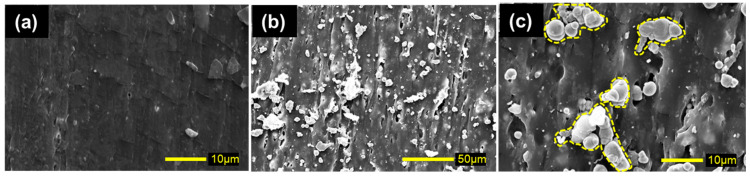
SEM photographs for (**a**) natural rubber (×2000) and magnetic rubber with a volume fraction of 0.11 in the absence of magnetic fields at magnifications of (**b**) ×500 and (**c**) ×2000. Aggregations of magnetic particles are indicated by yellow dot lines.

Figure 4 displays the SEM photographs for natural rubber and magnetic rubber with a volume fraction of 0.11. Aggregations of magnetic particles (indicated by yellow broken lines) were observed in photographs of magnetic rubber. However, many magnetic particles were also observed as primary particles. Therefore, the dispersibility of carbonyl iron is considered to be neither low nor high in the matrix of natural rubber. In fact, the storage modulus of the magnetic rubber at *ϕ* = 0.11 is 155 ± 10.1 kPa, which is slightly higher than that of the matrix (=150 ± 20.9 kPa). On the other hand, for aggregative barium ferrite, the storage modulus at the same volume fraction is 12.0 ± 0.75 kPa [40], reaching approximately twice that of the matrix (=5.78 ± 1.03 kPa). Thus, although carbonyl iron does not exhibit significant aggregation in natural rubber, its composite rubber demonstrates high nonlinearity. This may contribute to the reversible negative magnetorheological effect observed in this study. When a magnetic field is applied, the magnetic particles move and rearrange themselves into a chain-like structure. Even if this displacement is relatively large (*γ*~0.001), the strong nonlinear viscoelasticity of natural rubber allows the magnetic particles to change their position with low restoring force. The high adhesiveness between magnetic particles and natural rubber, combined with the strong nonlinearity of natural rubber, may enable this unique magnetic field response.

The magnetic field-dependent data must be valuable for elucidating the mechanism of the MR effect of the magnetic rubber; however, we mention it here only very briefly. General for MR elastomers, the hysteresis in the dynamic modulus is normally observed, and its mechanism seems to be very complex by coexisting the magnetostriction [48,49,50]. From the magnetic field dependence, it was found that the storage modulus decreased linearly with increasing the magnetic field below 250 mT and remained nearly constant at higher field, i.e., the change in the storage modulus at 500 mT is equal to that at 250 mT. This indicates that the destruction of the aggregation of magnetic particles was completed below 250 mT.

**Figure 5:**
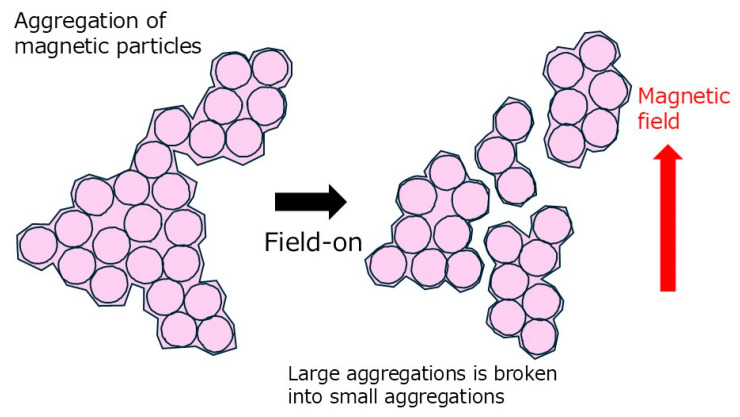
A possible mechanism of the negative and reversible changes in the storage modulus for magnetic rubbers.

Figure 5 displays a possible mechanism of the negative and reversible changes in the storage modulus for magnetic rubbers. In this magnetic rubber, aggregation of magnetic particles occurs to contribute to the elastic modulus. That is, the magnetic particles are in mutual contact. When a magnetic field is applied, the magnetic particles undergo rearrangement. This rearrangement here means primarily local rearrangement due to the rotation of magnetic particles, which is different from the macroscopic chain structure formation in conventional MR materials. Generally, magnetic particles rotate to achieve magnetic stability under a field, driven by effects such as the demagnetizing effect depending on particle shape or the easy axis of magnetization originating from crystal structure. This rotation is due to the magnetic interaction between magnetic momentum of magnetic particles and the magnetic field. Therefore, the rotation of magnetic particles may potentially cause the destruction of contact between magnetic particles. At maximum, the increase in elastic modulus due to the filler effect can be considered to be disappeared by the rotation of the magnetic particles.

## 3. Conclusions

Novel composite rubbers demonstrating negative and reversible changes in storage modulus were successfully developed first in this study. The storage and loss moduli of the composite rubber decreased when a magnetic field was applied, which is opposite to the magnetic response in the past. That is, the composite rubber exhibits sufficient rigidity to support objects at no magnetic fields; however, it softens only when a magnetic field is applied. This unconventional feature holds potential for a wide range of applications. Since the elastic modulus is variable, the scope of application is the same as conventional MR materials. However, when it is necessary to hold an object without applying a magnetic field, this magnetic rubber is suitable for the application. It is, for example, vibration absorption for engine mounts of electric vehicles. Magnetic rubber supports the engine when no magnetic field is applied; meanwhile, it can be softened only when a magnetic field is applied to shift the resonance frequency. This is because electric vehicles are required to conserve power as much as possible except when driving. With conventional MR materials, the electromagnet must be energized to keep the MR material in a hard state, i.e., it consumes a large amount of electric energy. The key challenge to be overcome in the future is durability. As the contact between magnetic particles within the magnetic rubber contributes to its elastic modulus, raising the issue of whether it can maintain its physical properties even after hundreds of thousands of operations. The mechanism for the negative and reversible magnetic response observed is unclear now; however, a strong interaction between magnetic particles and the rubber matrix, along with the strong nonlinear viscoelasticity of the rubber matrix, would be important in the mechanism of this unique phenomenon.

## 4. Materials and Methods

### 4.1. Synthesis of Magnetic Rubbers and Measurement of Degree of Swelling

Natural rubber, carbonyl iron particles with a diameter of 8.3 µm (CM Grade BASF SE., Ludwigshafen am Rhein, Germany), zinc oxide, stearic acid, anti-aging agent, and process oil were mixed using a 3 L test kneader (Suzuka Engineering Co., Ltd., Yokkaichi, Japan) at 100 °C for 7 min. A sulfur-based vulcanizing agent was added to the mixture and kneaded at 50 °C for 5 min, then stretched using an 8-inch open roll (HSU FENG IRON FACTORY Co., Ltd., Taichung, Taiwan). Subsequently, vulcanization was performed at 150 °C for 7 min using a press molding machine, yielding a sheet of magnetic rubber with a thickness of 1 mm. The concentration of magnetic particles is kept at 50 wt.%, corresponding to a volume fraction *ϕ* = 0.11. Rubbers without magnetic particles were also prepared in a similar manner with the magnetic rubber. The degree of swelling for samples was determined by the weight changes, *W*_wet_/*W*_dry_. The weight of the sample was measured at room temperature by an electronic balance (GH-200, A&D Co., Ltd., Tokyo, Japan) before and after immersion of the sample in acetone. The sample size was 20 mm in diameter and 1 mm thick. The densities of carbonyl iron and natural rubber were measured to be 7.5 g/cm^3^, 0.95 g/cm^3^, respectively, using electronic densimeter (MD-300S, Alfa Mirage Co., Ltd., Osaka, Japan).

### 4.2. Dynamic Viscoelastic Measurement

The dynamic viscoelastic measurement for the magnetic rubbers was performed using a rheometer (MCR301, Anton Par Pty Ltd., Graz, Austria) with electromagnetic system (PS-MRD) and a non-magnetic parallel plate (PP20/MRD). The frequency was kept at 1 Hz. The temperature was controlled at 20 °C using a constant temperature circulating device and it was monitored by a temperature sensor in the rheometer during the measurements. The sample size was 20 mm in diameter and 1 mm thick. In the rheological measurements, the strain range was from 10^−5^ to 1, and the initial normal force applied was approximately 0.3 N. A pulsatile magnetic field of 0 and 500 mT was applied for the switching experiment in Figure 3. The strength of the magnetic field at the sample stage was measured using a Gauss meter (TM-601, Kanetec Co., Ltd., Nagano, Japan). The magnetic field of 500 mT was generated by an excitation current of 3.0 A. The average values and standard errors of the storage and loss moduli were evaluated for three different samples obtained from one batch.

### 4.3. Scanning Electron Microscope Observations

To observe the dispersion of magnetic particles in magnetic rubbers, a cross-section of the magnetic rubber after Au deposition was observed using a scanning electron microscope (SEM) (JCM-6000 Neoscope, JEOL Ltd., Tokyo, Japan) at an acceleration voltage of 15 kV.

## Data Availability

The original contributions presented in this study are included in the article. Further inquiries can be directed to the corresponding author.
